# The Case Study of Nesfatin-1 in the Pancreas of *Tursiops truncatus*

**DOI:** 10.3389/fphys.2018.01845

**Published:** 2018-12-19

**Authors:** Claudia Gatta, Elena De Felice, Livia D’Angelo, Lucianna Maruccio, Adele Leggieri, Carla Lucini, Antonio Palladino, Marina Paolucci, Paola Scocco, Ettore Varricchio, Paolo de Girolamo

**Affiliations:** ^1^Department of Veterinary Medicine and Animal Production, University of Naples Federico II, Naples, Italy; ^2^School of Biosciences and Veterinary Medicine, University of Camerino, Camerino, Italy; ^3^Stazione Zoologica Anton Dohrn, Naples, Italy; ^4^Center for Advanced Biomaterials for Health Care, IIT@CRIB, Istituto Italiano di Tecnologia, Naples, Italy; ^5^Department of Science and Technology, University of Sannio, Benevento, Italy

**Keywords:** Nesf-1, pancreas, common bottlenose dolphin, glycemic state, type 2 diabetes mellitus

## Abstract

Nesfatin-1 (Nesf-1) is an anorexigenic peptide involved in the regulation of homeostatic feeding. Nesf-1 is expressed in the central nervous system and other organs, including pancreas, where it promotes the release of insulin from β-cells. This raises the possibility that Nesf-1 dysfunction could be involved in metabolic disorders, particularly in type 2 diabetes mellitus (T2D). Recently, it has been discovered that dolphins can be a natural animal model that fully replicates human T2D, due to its prolonged glucose tolerance curve and maintenance of a state of hyperglycemia similar to human T2D during fasting. This correspondence suggests that dolphins may be a suitable model for investigating physiological and pathological metabolic disorders. Here, we have characterized Nesf-1 distribution in the pancreas of the common bottlenose dolphin (*Tursiops truncatus*) and measured plasmatic levels of Nesf-1 and glucose during fasting and post-prandial states. The *Mediterranean Marine Mammal Tissue Bank* (MMMTB) of the University of Padova provided us with pancreas samples, derived from four animals, and plasma samples, collected before and after the main meal. Interestingly, our results showed that Nesf-1-immunoreactive cells were distributed in Langerhans islets, co-localized with glucagon in α-cells. Similar to humans, dolphin plasma Nesf-1 concentration doesn’t show a statistically significant difference when comparing fasting and post-prandial states. On the other hand, blood glucose levels were significantly higher before than after the main meal. Our data provide a comparative analysis for further studies on the involvement of Nesf-1 in mammalian metabolic disorders.

## Introduction

Nesfatin–1 (Nesf-1) is an 82 amino acid (aa) length polypeptide derived from calcium and DNA binding protein NUCB2 (NEFA/nucleobindin-2). Within the central nervous system (CNS), Nucb2 mRNA is present in different nuclei of the brain involved in feeding behavior. Until now, the mechanism of food intake inhibition via Nesf-1 has not been clarified. Intracerebroventricular administration of Nesf-1 and NUCB2 reduces food intake and body weight: Nesf-1 can act via melatonin system activation, independently of leptin pathway (Oh-I et al., 2006) or through the stimulation of neuropeptide Y (Stengel and Tache, 2013).

Outside the CNS, Nucb2 mRNA is mainly expressed in pancreas, gastric mucosa, duodenum, white adipose tissue and testis (Gonzalez et al., 2009; Shimizu et al., 2009; Stengel et al., 2009; Ramanjaneya et al., 2010; Garcia-Galiano et al., 2012; Kim et al., 2014). It has been shown that NUCB2/Nesf-1 is localized in β-cells of human and rodent pancreas, promoting the release of insulin (Gonzalez et al., 2011). This raises the possibility that impairment of Nesf-1 production and/or secretion could be involved in metabolic disorders, particularly in type 2 diabetes mellitus (T2D) (Gonzalez et al., 2009).

Although different animals have been used, a unique model species that fully complements human T2D has not been yet identified (Cefalu, 2006). Recently, it has been discovered that dolphins have a prolonged glucose tolerance curve and during fasting maintain a state of hyperglycemia similarly to human diabetes mellitus (Venn-Watson et al., 2010, 2012). The main difference with diabetic patients is that dolphins seem to alternate a physiological diabetes that goes off with fasting during the night. Therefore, common bottlenose dolphins might constitute a valid spontaneous model of T2D (Venn-Watson et al., 2010).

We decided to characterize Nesf-1 distribution in pancreas of the common bottlenose dolphin (*Tursiops truncatus*) and evaluate its pre- and post-prandial plasmatic levels. These data continue previous studies of our group on the neuropeptides of the gastro-entero-pancreatic system of marine mammals (Russo et al., 2012; Gatta et al., 2014). Our aim is to establish a morphological basis for understanding the mechanisms involved in the pathogenesis of mammalian endocrine diseases.

## Materials and Methods

### Animals and Sample Preparations

Pancreas and frozen plasma samples of two adult male and two adult female specimens of common bottlenose dolphin were retrieved from the Mediterranean Marine Mammal Tissue Bank (MMMTB) of the Department of Comparative Biomedicine and Food Science of the University of Padova. For morphological studies, pancreas paraffin embedded samples were used. Blood was originally drawn from veins of the tail flukes of four trained dolphins for routine veterinary medical controls. Plasma samples were collected at 10:00 a.m. (fasting state) and 17:30 p.m. (post-prandial state) in four different days, in February and August (two blood draws for each month).

Archival samples of male Wistar rat testis (Garcia-Galiano et al., 2012), stored in the Department of Veterinary Medicine and Animal Production (MVPA) of University of Naples Federico II, were used as positive controls. Ethic approval was not required neither for dolphins nor for rat samples because they were obtained, respectively, from the MMMTB and from the MVPA.

### Single Immunohistochemistry

Paraffin embedded sections were processed as reported also in Gatta et al. (2014). The primary antiserum employed is a polyclonal antibody raised in rabbit against Nesf-1 (1:500, Phoenix Pharmaceuticals, cat. No. H-003-24). Background was prevented by previous incubating sections in normal goat serum (NGS) [1:5, 30 min, room temperature (RT)]. Qualitative identification of antigens was detected with DAKO EnVision^TM^+ System, Peroxidase. Staining was completed by incubation with 3,3′-diaminobenzidine (DAB)+ substrate-chromogen (Sigma, St. Louis, MO, United States).

### Double Immunostaining

For the double Nesf-1/insulin immunostaining, the employed primary antisera were: anti-Nesf-1 (1:50, Phoenix Pharmaceuticals, cat. No. H-003-24) and anti-Insulin (1:50, Abcam, cat. No. ab7842). Sections were incubated overnight (ON) at 4°C with each primary antibody. After the incubation with the primary antibodies, the sections were rinsed several times and incubated, respectively, with Lissamine Rhodamine (1:1000, Jackson Immuno Research Labs, cat. No. 111-085-003 conjugated goat anti-rabbit) and fluorescein AffiniPure Donkey Anti-Guinea Pig IgG (H+L) (1:1000, Jackson Immuno Research Labs, cat. No. 706-095-148), for 2 h, RT.

For the double Nesf-1/glucagon-like peptide-1 (GLP-1) immunostaining, the employed primary antisera were: anti-Nesf-1 (1:50, Phoenix Pharmaceuticals, cat. No. H-003-24) and anti-GLP-1 (1:50, Santa Cruz Biotechnology Inc., cat. No. sc-7782). Sections were incubated at 4°C, ON with anti-Nesf-1, and 48 h with anti-GLP-1. After the incubation with the primary antibodies, the sections were rinsed several times and incubated, respectively, with Lissamine Rhodamine (1:1000, Jackson Immuno Research Labs, cat. No. 111-085-003 conjugated goat anti-rabbit) and 488-Affinipure donkey anti-goat (1:1000, Jackson Immuno Research Labs, cat. No. 705-545-147), for 2 h, RT.

For double staining, background was prevented by previous incubating sections in normal serum (1:5, 30 min, RT). Finally, sections were washed and mounted.

### Controls of Specificity

The specificity of immunohistochemical reactions was checked in repeated trials via pre-absorption of primary antibody Nesf-1 (H-003-24; Phoenix Pharmaceuticals) with homologous antigen Nesf-1 (1-45)/Nesf-1, N-terminal (Human) (003-24; Phoenix Pharmaceuticals) (up to 50 mg/ml antiserum in the final dilution). Positive controls were made by sections of rat pancreas (data not shown). Internal reaction controls were carried out by substituting primary antisera or secondary antisera with phosphate buffered saline or normal serum in the specific step (de Girolamo and Lucini, 2011).

### Image Acquisition

Fluorescent and light images were analyzed by Nikon Eclipse 90i. The digital raw images were optimized for image resolution, contrast, evenness of illumination, and background by using Adobe Photoshop CS5 (Adobe Systems, San Jose, CA, United States).

### Western Blot Analysis

Bottlenose dolphin pancreas and rat testis samples were processed as previously described (Gatta et al., 2014). The same blot membrane was stripped and re-probed against β-actin (A5060, Sigma, Sant Louis, MO, United States), used as internal marker. In the specific step, primary polyclonal antibody raised in rabbit against Nesf-1 (1:2000, Phoenix Pharmaceuticals, cat. No. H-003-24) was incubated ON at 4 °C. This was followed by incubation with the secondary goat anti-rabbit IgG (1:10000, Santa Cruz Biotechnology Inc., cat. No. sc-2004) for 1 h, RT.

### Nesf-1 Plasma Levels

Nesfatin-1 plasma concentration was measured with an enzyme immunoassay kit for Nesf-1 (1-82) (Human) (EK-003-26; Phoenix Pharmaceuticals Inc.) according to the manufacturer’s protocols.

### Glucose Plasma Levels

The glucose plasma levels were measured photometrically on the Abaxis VetScan VS2 chemistry analyzer.

### Statistical Analysis

Data were analyzed as comparison of media and any significant difference was determined at a significance level of 0.05 via the application of Student’s *t*-test, and Pearson correlation coefficient to indicate the extent to which Nesf-1 and glucose were linearly related.

## Results

### Morphological Analyses

**FIGURE 1:**
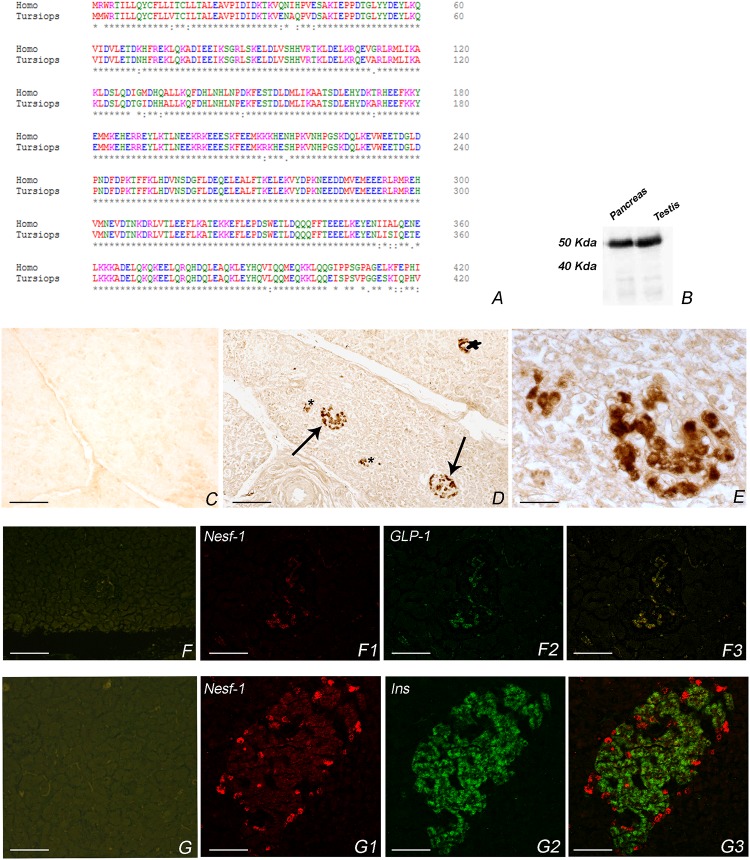
Nesf-1 in *Tursiops truncatus*. **(A)** Amino acid sequence alignment *T. truncatus* and *Homo sapiens*. Degree of conservation, indicated by asterisks, accounts for 95% (alignment was done with Clustal Omega http://www.ebi.ac.uk/Tools/msa/clustalo/). **(B)** Western blot in pancreas of bottlenose dolphin and rat testis showing an immunoreactive band of about 50 kDa. **(C–G3)** Transverse section of pancreas. **(C)** Negative control of anti-Nesf-1 in the pancreas of *T. truncatus.* **(D)** Nesf-1-icPlease cite “Figure 1 F1” inside the text. in pancreatic islet detected by single immunohistochemistry. Arrows indicate large islets; star indicate small islet; asterisks indicate exocrine components. **(E)** High magnification of **(D)**. **(F–F3)** Double immunostaining against anti-Nesf-1 and anti-GLP-1. **(F)** Negative control of anti-Nesf-1 and anti-GLP-1. **(F1)** Immunofluorescence of anti-Nesf-1 of double anti-Nesf-1/anti-GLP-1. **(F2)** Immunofluorescence of anti-GLP-1 of double anti-Nesf-1/anti-GLP-1. **(F3)** Double immunofluorescence of anti-Nesf-1/anti-GLP-1, showing co-localization in α-cells of islet. **(G–G3)** Double immunostaining against anti-Nesf-1 and anti-Insulin. **(G)** Negative control of anti-Nesf-1 and anti-Insulin. **(G1)** Immunofluorescenceof anti-Nesf-1 of double anti-Nesf-1/anti-Insulin. **(G2)** Immunofluorescence of anti-Insulin of double anti-Nesf-1/anti-Insulin. **(G3)** Double immunofluorescence of anti-Nesf-1/anti-Insulin showing different distribution pattern. Scale bars **C,D,F–F3** = 50 μm, **E** = 12 μm, **G–G3** = 25 μm.

Amino acid sequence of Nesf-1 of *T. truncatus* accounts for 95% of conservation to human Nesf-1 (Figure 1A). The employed polyclonal antibody is raised against human Nesf-1 (1–45). Furthermore, western blot analysis showed a Nesf-1 immunoreactive band at 50 kDa, as also observed in rat testis employed as positive control (Figure 1B). The internal marker β-actin was detected as a band of about 42 kDa (data not shown).

Nesfatin-1-immunoreactive cells (ic) were distributed mainly in the peripheral region of small islets. Additional cords of Nesf-1-ic were detected also in large islets or scattered in the exocrine component (Figures 1D,E,F1,G1).

To characterize the Nesf-1 distribution in pancreatic islets, we performed double immunofluorescence experiments against insulin and GLP-1. In the small islets, insulin-ic were located predominately in the central core while GLP-1 -ic were localized to the periphery; in larger islet cords of insulin-ic are interspersed with cords of glucagon ic-cells (Figures 1F2–G2). The merge results revealed a complete co-localization of Nesf-1 with GLP-1 in alpha cells of islets (Figure 1F3) whereas no co-localization of Nesf-1 and insulin positive beta cells was observed (Figure 1G3).

Negative controls of primary antibodies are in Figures 1C,F,G.

### Biochemical Analyses

#### Nesf-1 and Glucose Plasma Levels

**FIGURE 2:**
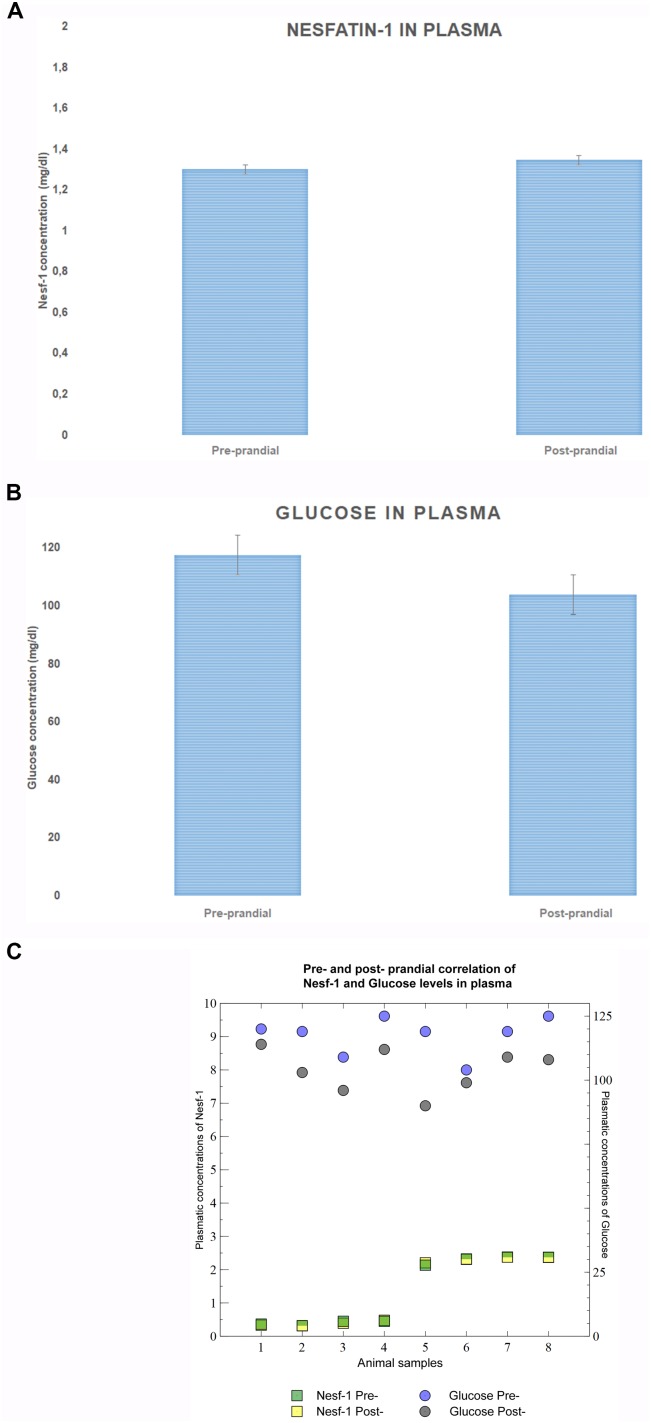
Nesf-1 and glucose plasma levels. **(A)** Plasma Nesf-1 concentrations didn’t show differences during fasting and post-prandial states. **(B)** Plasma glucose levels measured during fasting are significantly higher than those post-prandial states (*T-test p* < 0.01). **(C)** Pearson correlation coefficient was used to display a slight inverse linear correlation between Nesf-1 and glucose plasma concentration (*r_pre-prandial_* = –0.012; *r_post-prandial_* = –0.026).

Measurement of Nesf-1 plasma concentration revealed no statistically significant difference (*p*-value = 0.9328) between pre- and post-prandial states (Figure 2A). Glucose levels were significantly higher (*p*-value = 0.0039) in the fasting phase than after the meal (Figure 2B). Furthermore, Nesf-1 and glucose plasma levels displayed a slight inverse linear correlation (*r_pre-prandial_* = 0.82; *r_post-prandial_* = 0.007) (Figure 2C).

## Discussion

Here we describe for the first time the presence of Nesf-1 in the pancreas of common bottlenose dolphins. The pancreas of this species, as in most mammals including human, is organized into lobules, with many exocrine acini that produce enzymes necessary for digestion and variable numbers of randomly distributed Langerhans islets, containing glucagon (α-cells) and insulin (β-cells) producing cells (Colegrove and Venn-Watson, 2015; Cozzi et al., 2017).

Our data show that Nesf-1-ic are primarily distributed in the peripheral region of small islets of Langerhans and appear colocalized in glucagon α-cells of pancreas. Previous studies suggested that NUCB2/Nesf-1 is expressed in the endocrine islets of pancreas in rodents (Gonzalez et al., 2009; Stengel et al., 2009) and humans (Foo et al., 2010). Furthermore, it was established that NUCB2/Nesf-1 and insulin are co-localized in β-cells in rodents (Gonzalez et al., 2009) and humans (Foo et al., 2010) suggesting a role in the regulation of glucose homeostasis, particularly promoting the release of insulin (Figlewicz and Benoit, 2009), in concentration-dependent manner (Nakata et al., 2011).

The co-localization of Nesf-1 with GLP-1 we reported in bottlenose dolphin pancreas can be correlated with the peculiar glycemic state of this animal. In addition, our results confirm high serum glucose levels during the fasting state, as already demonstrated (Venn-Watson and Ridgway, 2007), possibly due to the low carbohydrate diet and high demands for cerebral glucose needs. Thus, we hypothesize that Nesf-1 could sustain GLP-1 secretion to maintain high blood sugar levels. In fact, Venn-Watson et al. (2013) showed that dolphins in fasting state had higher levels of glucagon compared to those in post-prandial state (170 and 152 pg/ml, respectively). A recent study (Riva et al., 2011) described a positive correlation between NUCB2 and glucagon gene expression in human, suggesting that Nesf-1 is secreted by β-cell as a response to glucose, and could act via paracrine mode to stimulate glucagon secretion. Riva’s data asserted that Nesf-1 is a stimulator of both insulin and glucagon secretion, hypothesizing different regulatory mechanisms for the two hormones. An analogous regulation can be hypothesized in dolphins. However, future studies are necessary to unravel the mechanism behind biological effects of Nesf-1, including the challenge of identification of the receptor.

Remarkably, when evaluating plasma Nesf-1 levels in common bottlenose dolphins, we do not observe significant differences between fasting or post-prandial states, similarly to data reported in humans (Li et al., 2010). Nesf-1 plasma levels displays a very slight inverse correlation to glucose plasmatic levels, therefore the regulatory mechanisms regulating the blood circulating glucose and Nesf-1 levels requires further investigations.

Overall, the physiological similarities between primates and cetaceans support a shared drive for common glucose metabolism. Only primates and cetaceans have red blood cells ‘extraordinarily’ permeable to glucose (Craik et al., 1998) and share high encephalization quotient (EQ) (Jerison, 1973). These latter relevant aspects can be the key to explain high metabolic demand of the primates and cetaceans’ large brains (Goodwin, 1956).

## Conclusion

Our results add new data on the presence and distribution of Nesf-1 in the pancreas of common bottlenose dolphins. These reports highlight the potentiality of this animal species as natural model to unveil the mechanisms involved in human T2D. Further research is needed to fully understand the regulatory mechanisms underlying the glycemic state in dolphins.

## Availability of Data and Materials

All data generated or analyzed during this study are included in this published article.

## Ethics Statement

For the present Please confirm if the Ethics Statement included here is fine.study pancreas and frozen plasma samples of two adult male and two adult female specimens of common bottlenose dolphin (*Tursiops truncatus*) stored at the Mediterranean Marine Mammal Tissue Bank (MMMTB) of the Department of Comparative Biomedicine and Food Science of the University of Padova (http://www.marinemammals.eu) were used. The MMMTB is a recognized CITES institution (IT020) that stores tissues removed from stranded animals or from marine mammals who died in captivity and were referred for postmortem.

## Author Contributions

PdG and LDA conceived and supervised the entire study. CG, LM, and AL collected the data. LDA and AP analyzed and interpreted the data. EDF drafted the manuscript. AL and LDA made the figures. CL, PS, MP, and EV critically revised the manuscript. All authors have approved the submitted manuscript.

## Conflict of Interest Statement

The authors declare that the research was conducted in the absence of any commercial or financial relationships that could be construed as a potential conflict of interest.

## Abbreviations

a.m.: ante meridiem
CNS: central nervous system
g: relative centrifuge force
GLP-1: glucagon-like peptide-1
h: hour
ic: immunoreactive cells
kDa: kilodalton
min: minute
Nesf-1: Nesfatin-1
NUCB2: NEFA/nucleobindin-2
ON: over-night
p.m.: post meridiem
rpm: revolution per minute
RT: room temperature
T2D: type 2 diabetes mellitus
V: Volt
W: Watt
α: alpha
β: beta
